# Health Characteristics and Treatment Patterns of VHA Veterans Diagnosed With PTSD in Later Life

**DOI:** 10.1093/geroni/igaa057.979

**Published:** 2020-12-16

**Authors:** Linh Dang, Jenefer Jedele

**Affiliations:** Veterans Health Administration, Ann Arbor, Michigan, United States

## Abstract

Older adults may exhibit symptoms of Post-Traumatic Stress Disorder (PTSD) after a traumatic event, even if they do not meet the criteria for a full diagnosis. This sub-clinical PTSD affects 7-15% of older adults and is associated with elevated depressive symptoms, suicidal ideation, and poorer physical health. Individuals with sub-clinical PTSD often experience worsening symptoms, resulting in a full diagnosis of PTSD, albeit late onset. This evaluation informs the Veterans Health Administration (VHA)’s understanding of the developmental course of PTSD in older Veterans. VHA administrative data were used to examine health characteristics and service utilization in the five years before and five years after the initial VHA documentation of PTSD. We identified a cohort of Veterans (n=27,610), alive and with at least 1 encounter in all evaluation years, with a first PTSD diagnosis documented between ages 50-59, a cohort outside of the average age of diagnosis, but before Medicare eligibility. We compared periods before and after diagnosis across different ages of first diagnosis (50-54, 55-59). Veterans diagnosed at later ages (55-59) had a greater number of mental and physical health conditions. Increasing VHA use preceded a PTSD diagnosis in both groups, but the increase was steeper among those diagnosed at 55-59. Future analyses will compare these patterns to those of Veterans diagnosed at younger ages. Findings from this work may provide a profile of persons at risk for late-life PTSD for use in targeting interventions earlier to reduce the risk of developing worsening PTSD symptoms in late life.

